# Mastering DNA Content Estimation by Flow Cytometry as an Efficient Tool for Plant Breeding and Biodiversity Research

**DOI:** 10.3390/mps6010018

**Published:** 2023-02-12

**Authors:** Maria Fomicheva, Elena Domblides

**Affiliations:** Federal State Budgetary Scientific Institution Federal Scientific Vegetable Center (FSBSI FSVC), VNIISSOK, 143072 Moscow, Russia

**Keywords:** acquisition settings, C-value, genome size, nuclear isolation buffer, plant sciences, ploidy

## Abstract

Flow cytometry gives a unique opportunity to analyze thousands of individual cells for multiple parameters in a course of minutes. The most commonly used flow cytometry application in plant biology is estimation of nuclear DNA content. This becomes an indispensable tool in different areas of plant research, including breeding, taxonomy, plant development, evolutionary biology, populational studies and others. DNA content analysis can provide an insight into natural ploidy changes that reflect evolutionary processes, such as interspecific hybridization and polyploidization. It is also widely used for processing samples with biotechnologically induced ploidy changes, for instance, plants produced by doubled haploid technology. Absolute genome size data produced by cytometric analysis serve as useful taxon-specific markers since genome size vary between different taxa. It often allows the distinguishing of species within a genus or even different subspecies. Introducing flow cytometry method in the lab is extremely appealing, but new users face a significant challenge of learning instrument management, quality sample preparation and data processing. Not only is flow cytometry a complex method, but plant samples have unique features that make plants a demanding research subject. Without proper training, researchers risk damaging the expensive instrument or publishing poor quality data, artifacts or unreproducible results. We bring together information from our experience, key papers and online resources to provide step by step protocols and give a starting point for exploring the abundant cytometry literature.

## 1. Introduction

DNA content analysis by flow cytometer is gradually replacing classic chromosome counting and other methods since it allows analysis of a large number of cells with unprecedented speed and precision. It is extremely useful when a large number of samples has to be processed. For instance, it is used for various plant breeding applications, including testing regenerants produced by doubled haploid technology, breeding of polyploid species or cultivars, somatic hybridization and detection of tissue culture soma-clonal variation [1,2]. DNA content can be measured for systematics purposes to distinguish closely related species. It can also be used for studies of interpopulation ploidy variation and interspecific hybridity and polyploidy occurrence during evolutionary process. It can be useful for classification of plants lacking organs necessary for species determination, i.e., reproductive organs [3]. It can be applied for studying patterns of endopolyploidy in different plant organs at different developmental stages. High resolution flow cytometry analysis can detect aneuploidy with single chromosome or even chromosome arm differences [1,4,5].

Flow cytometry can be a powerful tool in plant biology labs with different focuses. However, plant cells have a list of features that has to be taken into account for successful cytometric analysis. With the exception of single cell algae, plants are multicellular organisms. Single cell isolation is complicated by cell walls surrounding each cell. Plant cells also have irregular shape and an acentric position of the nucleus. This makes a laser beam interact differently with different cells, which negatively affects the accuracy and reliability of the analysis. Besides, plant cells often approach or exceed the size of the flow cytometer nozzle at least in one dimension [5]. For studying genome size, these issues can be bypassed by isolation of nuclei in a hypotonic buffer [6]. It is possible to study intact cells by flow cytometry in case of pollen grains, sperm cells and microspores if they meet the requirements described above. Protoplasts are also suitable for flow cytometry since they are devoid of cell walls and can be analyzed as a single cell suspension [7].

Another significant complication in plant material analysis is a large number of secondary metabolites that can interfere with staining and/or produce autofluorescence. Secondary metabolite content can vary between different cultivars. It can also fluctuate between plants of different age or plants grown in different seasons and habitats. To reduce the differences in secondary metabolite content between individuals, it is preferable to take samples of the same age grown under similar conditions [5]. Addition of different buffer supplements can decrease the negative effects of secondary metabolites [8,9].

Starting cytometric analysis of plant samples is also complicated by the fact that there are less flow cytometry specialists in this field compared to biomedical research. Flow cytometry core facilities and users, as well as cytometer company representatives, are mostly familiar with analysis of animal cells. However, plant samples have a drastically different sample preparation and acquisition setup. With limited availability or absence of experienced specialist help, literature perusal becomes critical for performing and troubleshooting in plant cytometry. The large number of factors required for cytometry success can extend the time needed to obtain quality results to many months.

This paper is aimed at shortening the path of a literature search, as well as of the trial and error process. We place emphasis on critical details learned from key papers and on experience of honing the skills necessary to obtain quality results in different plant samples.

## 2. Experimental Design

The success of flow cytometric analysis depends on multiple factors, from tissue and buffer choice to flow cytometer settings (Table 1). Different plant tissues can be chosen for analysis, with the preference given to young leaves (Table 1). To test sample ploidy or DNA content, the sample and reference standard with known DNA content must be analyzed during the same instrument run [8]. The reference can be external or internal, for the same or a different species depending on the application. For instance, for doubled haploid selection the aim is to test the regenerant plant ploidy. A relative measurement of the genome size in comparison to the external reference is sufficient. A seed-derived plant from the same species can be used as a reference [10]. To exclude the possibility that the reference plant has any large-scale genome dissimilarities, it is better to use a plant from the same population/variety or a variety with confirmed DNA content.

Internal reference standard means that the reference and sample tissue are chopped and mixed in a sandwich like manner for simultaneous analysis [8]. An internal standard from the same species is used for aneuploidy detection to obtain higher precision measurement. A reference plant from a different species with known DNA content is required for absolute DNA content measurement. There is a list of cultivars with known genome size that are often used as standards [8,11,12]. *Ficus benjanina* or other indoor plants with confirmed DNA content can be used as a reference standard since they can be readily available for analysis [13].

Plant tissues are mechanically disrupted by razor chopping or bead beating in a hypotonic buffer to release nuclei and stain them with a DNA-specific fluorescent dye (Table 1). There is a choice of different hypotonic buffer recipes that allow gentle isolation of nuclei (Supplementary Tables S1–S5). Joao Loureiro and colleagues compared different buffers on different species of plants. They listed the best to worst buffers for the isolation of nuclei: LB01 and Otto were generally the best buffers, Galbraith buffer had intermediate results and Tris-MgCl_2_ buffer gave the worst results [14]. Based on these data, other buffer options were developed and can be tried [9]. Several buffers should be tested for a new species in order to choose the best option [14]. In addition, the authors tested the buffers without reductants, which can significantly improve the result quality. We provide information on reductant use in Section 3.1.3.

**Table 1:** Key factors for flow cytometry setup and troubleshooting.

Variables	Difficulties	Options	References
**Tissue choice**	High secondary metabolite content, sturdy tissues, high level of endopolyploidy	Young leaves are preferred. It is possible to use other tissues, callus and seeds.	[1,15]
**Lysis buffer**	Poor isolation of nuclei, too much debris	Otto, LB01, Galbraith, Tris-MgCl_2_ and other buffers	[9,14]
**Buffer supplements**	Secondary metabolites perturb DNA staining. Loss of reproducibility	Ascorbic acid, β-mercaptoethanol, sodium metabisulfite, Polyvinylpyrrolidone (PVP) supplements	[16]
**Nuclei isolation**	Poor isolation of nuclei, too much debris	Adjust chopping or bead beating time and vigor, use a new razor blade	[6,8,10,17]
**Cytometer setup and maintenance**	Results are not reproducible, poor peak quality, no peaks	Cytometer startup, quality control and cleaning	Flow cytometer manufacturer’s guidelines
**Acquisition settings**	Population of nuclei is not found, too much non-nuclear debris is visible	Adjust gain, threshold and plot properties	[18], Flow cytometer manufacturer’s guidelines
**Data processing**	Poor peak CV (coefficient of variation). High DNA content/ploidy variation between experimental repeats.	The instrument deep clean and/or de-bubbling is needed. Different buffers or antioxidants should be tried.	The references above

The isolated nuclei are filtered to reduce debris content and stained with a DNA-specific fluorescent dye. The sample is loaded in the flow cytometer that has to be set up before sample preparation is started (Table 1). The instrument setup and maintenance, as well as acquisition settings, are usually not covered in publications, but these steps are essential for obtaining reliable data. It can become a serious limitation for flow cytometry implementation in the lab and should not be omitted. The acquired data are analyzed by the same software that is used for acquisition or by a different program, for instance, free Flowing software (see Section 3.4).

### 2.1. Materials

1. Plant sample and reference standard tissue (for details refer to Section 3.1.1).
2. Lysis buffer components (for buffer recipes refer to Supplementary Tables S1–S5).
3. NaOH and HCl for adjusting pH.
4. PI stock solution (Sigma-Aldrich, St. Louis, MO, USA; Cat. no. P4170). Dilute powder in MilliQ or dH_2_O to stock concentration 1 mg/mL. Take time to fully dissolve PI. CAUTION: MUTAGEN, TOXIC. Handle with gloves, do not inhale. Store in a dark location at 4 °C.
5. DAPI stock solution (Sigma-Aldrich, USA; Cat. no. D9542). Dilute powder (stored at room temperature) in MilliQ or ddH_2_O to stock concentration 1 mg/mL. Store the stock solution in a dark location at −20 °C.
6. RNase A, DNase and protease-free (10 mg/mL) (ThermoFisher Scientific, Waltham, MA, USA; Cat. no. EN0531).
7. Ascorbic acid.
8. Sodium metabisulfite.
9. β-mercaptoethanol (Sigma-Aldrich, USA; Cat. no. M6250). CAUTION: TOXIC.
10. PVP with different molecular weights (10–40K) e.g., PVP10 (Sigma-Aldrich, USA; Cat. no. PVP10) or PVP40 (Sigma-Aldrich, USA; Cat. no. PVP40).
11. Sodium azide.
12. Calibration beads recommended by the cytometer manufacturer (e.g., CytoFLEX Daily QC fluorospheres, Beckman Coulter, Brea, CA, USA; Cat. no. B53230).
13. Sheath fluid recommended by the cytometer manufacturer (e.g., CytoFLEX Sheath Fluid, Beckman Coulter, USA; Cat. no. B51503) or homemade options described in Section 3.2.1.
14. Decontamination solution, e.g., diluted Contrad 70: H_2_O = 1:1 (Contrad 70, Beckman Coulter, USA; Cat. no. 81911).
15. Cleaning solution (e.g., FlowClean Cleaning Agent, Beckman Coulter, USA; Cat. no. A64669).
16. Bleach.

### 2.2. Equipment and Disposables

- Flow cytometer with a software provided by a manufacturer. This has to have a light source with the fluorochrome excitation wavelength—535 or 358 nm for PI or DAPI, respectively (e.g., CytoFLEX Beckman Coulter, USA, Cat. no. B53017).
- pH-meter.
- pH calibration solutions.
- Tissue homogenizer (e.g., TissueLyser, Qiagen, Germany)—optional.
- Plastic Petri dishes (60 or 100 mm). Plastic dishes are preferred since razor blades remain sharp longer.
- Razor blades
- A 20–50 µm mesh or a pre-separation filter (e.g., 30 µm filter, Miltenyi Biotec, 130-041-407). Can be reused if immediately washed with tap water and rinsed with dH_2_O.
- Flow cytometry tubes (e.g., Beckman Coulter, USA; Cat. no. 2523749)
- Tube racks
- Ice container or ice pads.
- Micropipette and suitable tips (10, 200, 1000 µL).
- Nitrile gloves of appropriate size.
- A computer with flow cytometry software e.g., Flowing software (free option) or a commercial software.

## 3. Procedure and Expected Results

### 3.1. Sample Preparation

#### 3.1.1. Sample and Reference Standard Tissue Collection

Pick fully expanded young leaves with no signs of disease or parasite infestation. Very young leaves might have many fluorescence inhibitors, such as anthocyanins [19]. On the other hand, old tissues can undergo rounds of endoreduplication, as well as accumulate infections and secondary metabolites. Etiolated leaves or leaves grown *in vitro* have lower secondary metabolite content that improves data quality.

Other tissues/organs can be used if young leaves are not available or if tissues/organs other than leaves are a subject of research. However, they should be analyzed with great caution because they can contain a high proportion of endopolyploid cells. Highly endo-polyploid tissues might have a low or even absent G_0_/G_1_ peak. In this case, G_2_ peak can be mistaken for G_0_/G_1_. In addition, some plants can undergo incomplete endoreduplication. It was also observed that DNA accessibility for staining can vary between nuclei of different ploidy which can lead to misinterpretation of results [1].


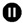
 PAUSE STEP. Fresh sample and reference standard tissues are preferred, but it is possible to store them at 4 °C wrapped in a moistened paper in a Petri dish for up to several days (a preliminary test is needed to make sure it works for the species of interest). 

It is also possible to use herbariums for cytometric analysis. It is crucial to use 5–10 times greater quantities of dehydrated material for chopping to record a sufficient number of nuclei. Desiccation has to be fast since tissue browning and decaying is detrimental for nuclear integrity. It can be possible to perform cytometric analysis on herbariums that are several months and sometimes even several years old. However, the best results are achieved on plants that were dehydrated within several weeks prior to analysis. For detailed protocol refer to [15]. Some authors also succeeded in using fixed or frozen material for cytometric analysis [20,21,22].

#### 3.1.2. Lysis Buffer Preparation

- Prepare the buffer based on the recipe and adjust pH (Supplementary Tables S1–S5). Different buffers should be tested for a new species.
- Prechill to 4 °C.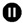 PAUSE STEP, Store the buffer in the fridge (except for Otto II, which is stored at room temperature). To prevent contamination, this is sterile filtered after preparation and aliquot. Galbraith buffer aliquots can be frozen for future use (do not refreeze) [8]. The addition of preservatives such as sodium azide can also be considered.

#### 3.1.3. Buffer Supplement Addition

Buffer supplements are added immediately before isolation of nuclei (Supplementary Table S6). Keep the buffer on ice.

- Immediately before measurements add RNase at 50 µg/mL.
  RNase addition might be omitted for some samples, although it should be tested for each case separately. In general, leaves have lower RNA content, but meristem and seed cells are abundant with RNA [19].
- Immediately before measurements, add an antioxidant such as 0.2 mg/mL ascorbic acid, 10 mM sodium metabisulfite, 15 mM β-mercaptoethanol (CAUTION: TOXIC). PVP (*v*/*v* 1–2%) with different molecular weights (10–40 K) can be used for tannin binding [16]. 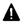 CRITICAL STEP. If sample darkening is observed (for instance, beet leaves turn brown without antioxidants within minutes), the addition of antioxidants becomes a key step for reducing negative secondary metabolite effects, i.e., uneven staining leading to unreproducible results.

#### 3.1.4. Addition of a DNA-Specific Fluorescent Dye

A DNA-specific fluorescent dye can be added at this point. In this case isolation and staining of nuclei will be performed simultaneously. Some authors prefer to add the dye after isolation of nuclei if a large number of samples is processed. This allows reducing the difference in staining time between the first and the last sample. Besides, addition of the dye after the nuclei are isolated can be preferred when PI is used to stain the nuclei, because chopping of plant material with PI, which is a toxic compound, can contaminate the working area.

- Add the fluorescent dye to the lysis buffer. 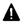 CRITICAL STEP. There should be saturating dye concentration in the solution to prevent variation in the dye uptake due to different numbers of nuclei in different samples. The final concentration for Propidium iodide (PI) is 50 µg/mL (CAUTION: MUTAGEN). 4′,6-diamidino-2-phenylindole (DAPI) is used at 4 µg/mL concentration. Other DNA-specific fluorescent dyes can also be considered [4].

#### 3.1.5. Isolation of Nuclei

- Put a small piece of plant tissue (5–50 mg) on a Petri dish. Add an ice-cold buffer (300 to 1000 µL). Keep the sample on ice.
- Mechanically disrupt tissues to isolate nuclei.

Quickly chop the tissue with a razor blade. 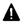 CRITICAL STEP. The blade must be sharp to cut the tissue in fine slices because squeezing or crushing damages the nuclei. Keep chopping in one direction like cutting bread and do not go back and forth to avoid damage to the nuclei. Use less material for chopping, since more tissue produces more debris. Thick leaves might need less chopping. For instance, fine chopping of cabbage leaves produces a lot of debris, but cutting the leaf a few times is enough for peak quality. 

The bead beating method can be tried instead of razor chopping to process multiple samples at the same time. Plant tissue and zirconia/silica/tungsten beads are placed in a tube. Otto I lysis buffer is added. Then the tissues are disrupted in a tissue homogenizer. The author of the bead beating method used FP120 FastPrep Disrupter (Savant Instruments) at 4.0–6.5 m/c for 45 s [17]. Other authors disrupted tissues at 25 Hz for 24 s in a TissueLyser (Qiagen, Germany) [10]. Then filtering and staining is done as usual.

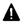
 CRITICAL STEP. Filter the sample through a 20–50 µm nylon mesh or a filter (the smaller pore size can be used for smaller nuclei). This is a required to prevent the cytometer clogging.
- OPTIONAL STEP. Recalcitrant samples can be gently centrifuged and resuspended in a fresh buffer to remove secondary metabolites.

#### 3.1.6. Sample Incubation

- OPTIONAL STEP. Add a DNA-specific fluorescent dye to the sample if it was not added to the buffer prior to isolation of nuclei (Section 3.1.4).
- Incubate the sample on ice from a few minutes to 1 h. Some samples should be analyzed immediately after preparation to avoid sample oxidation. 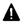 CRITICAL STEP. Incubation time has to be determined for each species experimentally. If incubation time is too short, the staining of nuclei will be incomplete. If incubation time is too long, staining can decline due to secondary metabolites.

### 3.2. Instrument Setup and Maintenance

It is highly recommended to learn the principles of flow cytometer work from the literature or online tutorials before planning experiments [23,24]. This helps to understand how data are produced and how to troubleshoot arising problems. The instructions presented below provide information for instrument startup logic summarized from recommendations for different instruments (CytoFLEX by Beckman Coulter, Attune NxT by Invitrogen, BD FACSCalibur by BD Biosciences). Refer to the cytometer manual for instrument-specific details. Correct instrument setup prior to measurements is crucial for obtaining accurate results and maintaining the system in working condition.

#### 3.2.1. Instrument Startup

- Record the session in the instrument use journal or online calendar.
- Refill Sheath fluid container (for some machines this has to be done on working machines in a standby position).

There are commercially available and homemade sheath fluid options. There should be no bubbles or any particles (dust, bacteria, algae, etc.) in sheath fluid because it will perturb fluidics work or clog the system. One of the options is PBS with addition of sodium azide (CAUTION: TOXIC) as a preservative to prevent bacterial growth. However, for some cytometers, saline-based sheath fluid is not recommended since it can damage instrument components (CytoFLEX, Beckman Coulter). The alternative option is Milli-Q or dH_2_O filtered through a 0.22 µm filter. Water works for analyzers because the sheath and sample core stream do not mix. Water cannot be used on FACS machines when living cells are collected after analysis for further culturing, since cells will be mixed with sheath fluid.

- Empty waste container (for some machines this has to be done on working machines in a standby position).

Add a small amount of bleach solution according to manufacturer’s instructions to an empty waste container. It deactivates biohazardous samples and dangerous chemicals (e.g., PI) that are collected during the run.

The instrument will signal during the run if waste removal or sheath fluid refill is needed again.

- Switch uninterruptible power supply if applicable (recommended if power shortage is possible in the area).
- Switch the cytometer button on.
- Switch the computer on.
- Start the flow cytometry program.
- Run the System Startup Program (or Priming/Initialization). Follow the instrument instructions.

During instrument startup lasers warm up, pumps initialize and instrument fluidics are primed. The system also informs the user if any problems are found.

#### 3.2.2. Instrument Quality Control (QC) 

Depending on the machine, this function can be named QC, Calibration Beads, Performance test, etc. 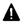 CRITICAL STEP. Perform QC every time the instrument is used to ensure accuracy and sensitivity of the instrument.

- Start QC/Standardization function in the cytometer program or a separate calibration software (for BD Biosystems cytometers).
- Prepare the instrument manufacturer recommended fluorescent beads. Vortex the beads and add them to distilled water or bead dilution buffer according to manufacturer’s instructions.
- Vortex or tap the tube with the beads and load them into the tube holder.
- Enter lot-specific target values for the calibration beads.
- Run the quality control.

After QC successful completion, take the tube out. Fluorospheres can be stored sealed in a dark location at 4 °C for up to 5 days (this may differ for different beads).

If QC fails, Prime, Daily Clean and Deep Clean should be performed. If QC fails again, the cytometer company representative should be contacted.

Perform a daily clean to remove fluorescent beads from the system.

#### 3.2.3. Daily Clean

Daily clean (or SIP sanitize and Rinse on some machines) is performed according to manual instructions during system startup and shutdown, between the users, between different batches of samples, or after a sample that could clog the system.

#### 3.2.4. Daily Shut Down

It is crucial to finish the instrument work correctly, otherwise there is a risk of system clogging due to microbial contamination, as well as sample, fluorochrome or salt buildup.

- Perform Daily Clean (or a specialized Shutdown function for some instruments) according to the manual instructions.
- Empty the waste bottle and add bleach, refill sheath fluid.
- Exit the program and switch off the system.

#### 3.2.5. System Decontamination

- Regularly perform System Deep Clean (or System Flush, Decontaminate System function for some machines) to remove debris and microbial growth.

Follow manual instructions on the procedure details and frequency. Flow cytometer is recommended to be used regularly in order to maintain proper cytometer fluidics operation. If the instrument was not in use for more than 10–14 days, perform Deep Clean program before starting measurements.


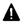
 CRITICAL STEP. Deep Clean can be carried out if acquisition problems occur. If populations are drifting, their amplitudes are decreasing and CV is increasing, it is possible that the flow cell needs decontamination, or bubbles are present in the system. Run Priming and Deep Clean.

- Sheath fluid and waste bottle should be decontaminated regularly according to manufacturer’s guidelines.

### 3.3. Acquisition Settings and Plot Setup

#### 3.3.1. Creating Experiment from a Template

- The settings from a previous experiment can be used: the experiment can be saved as a template. Then create a new experiment from template. This will have all the settings from the previous experiment. Alternatively, open a pre-existing experiment, start recording new data, delete data from the previous experiment, save under a new name.
- If there is no template available, follow the instructions below.
- Prepare the samples (see the protocol in Sample preparation section).

#### 3.3.2. Creating a New Experiment 

If the user has not performed a flow cytometry experiment before or samples of interest were not tested for flow cytometry in the past, it is advised to use a “simple” sample for acquisition settings and control of sample preparation procedure. For instance, Pisum sativum leaves can be used for this purpose.

**Figure 1:**
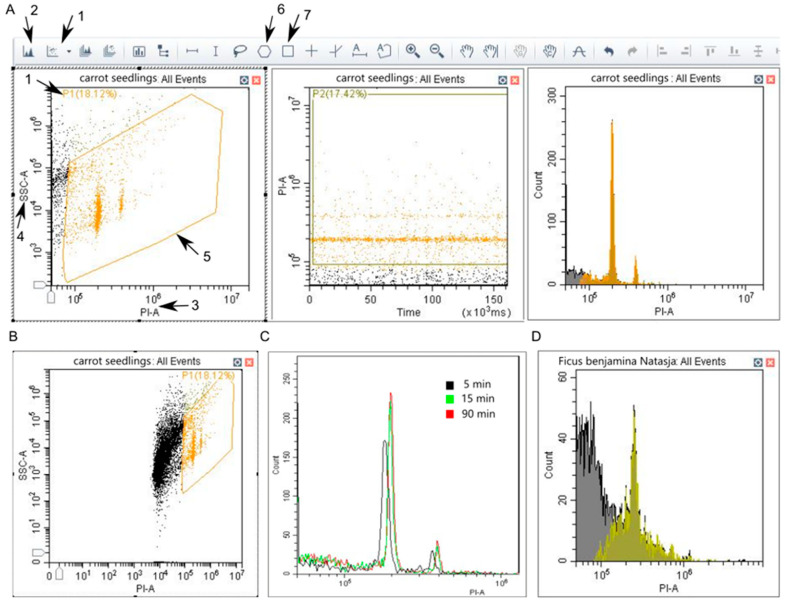
Flow cytometer acquisition settings for DNA content analysis. (**A**). Create two dot plots and a histogram by pressing (1) and (2), respectively. Adjust axes by pressing on them and selecting PI-A (3) and SSC-A (4). Change axes to Time and PI-A for the second plot. Select PI-A for X axis of the histogram. The population of interest (5) can be selected by polygonal (6) and rectangular (7) tool. (**B**). Cytometric data before adjusting plot properties. A large population of debris is observed. The target population (P1) makes up less than 20% of recorded particles. (**C**). Carrot seedling nuclei stained with PI for 5 (black), 15 (green) and 90 (red) minutes. (**D**). Example of poor isolation of nuclei. Nuclei are partially masked by excessive debris population.

- Create a New Experiment, select a location for the experiment file.
- Select channels that will be used. Use detection from a single laser that is closer to the dye excitation wavelength (535 or 358 nm for PI or DAPI, respectively).
- No compensation is needed since the single fluorescent channel is used.
- Create two dot plots and a histogram (Figure 1A).
- Adjust Acq. Settings.

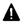
 CRITICAL STEP. To find the population of interest, adjust gain, threshold and plot properties (min, max, log/linear scale).

- Set Gain settings as follows: FSC = 81, SSC = 20, PI = 25.
- These settings work well for Cytoflex machine by Beckman Coulter [18]. Adjustments might be needed for a different machine. Change gain values to move most events to the center of the plot.
- Set Threshold: select fluorescent channel as a primary threshold, Manual, 10,000, Area. Further adjust the threshold if results are not optimal.
- These settings are different from typical settings used for animal cells. Triggering is carried out on the fluorescent channel instead of FSC and SSC because a large proportion of the particles in a sample is debris and organelles that need to be eliminated from the analysis. The threshold cuts off non-fluorescent objects.
- Adjust plot properties to better visualize the nuclei: right click the plot and choose properties.
- Set up min values to further cut off debris and organelles. 10,000–50,000 and 160 for X and Y, respectively, can be used as a starting point.

If populations of interest are not visible, lower threshold and min values (Figure 1B). Gradually increase the cutoff for best visualization of the nuclei. The scale can be set to logarithmic format to find populations of interest, since it fits a larger range of events on the axis. The logarithmic scale should not be used for genome size estimation on older machines, but for newer cytometers data can be displayed on linear or logarithmic scale, since underlying data will remain linear. Log scale can be useful if different samples significantly vary in genome size or samples with extensive endoreduplication are studied [1].

The same settings work for most samples. If the genome is too large or plant cells undergo multiple rounds of endoreduplication, the settings can be adjusted accordingly to fit the nuclei into the graph.

- On the acquisition window Choose slow speed for better data quality.
- Adjust Events to Record so that the number of nuclei for each G_0_/G_1_ peak is ≥1000 nuclei [1].
- Tap the sample to mix and load it into the cytometer tube holder.
- Press Run to observe the events; press Record to save data from particles running through the cytometer.
- Do not change acquisition settings between samples.
- If a new species or tissue is tested, run a preliminary experiment with a couple of samples to test different staining times. Run the same sample at different timepoints to check when the DNA is saturated with the dye (Figure 1C).
- If DNA peaks are partially masked by debris or absent (Figure 1D), try to repeat the isolation of nuclei. If this does not help, a different buffer, antioxidants or a different plant tissue can be tried.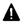 CRITICAL STEP. If external standardization is used, run the reference standard after every 10–20 samples to check for the DNA peak position fluctuation due to instrument heating [1,16].

### 3.4. Data Processing in a Cytometry Program

#### 3.4.1. Visual Data Assessment by Histogram Overlay

This can be sufficient for practical purposes, such as regenerant ploidy assessment for doubled haploid breeding program [10].

- Overlay the reference standard and unknown sample histograms if external standard was used.

**Figure 2:**
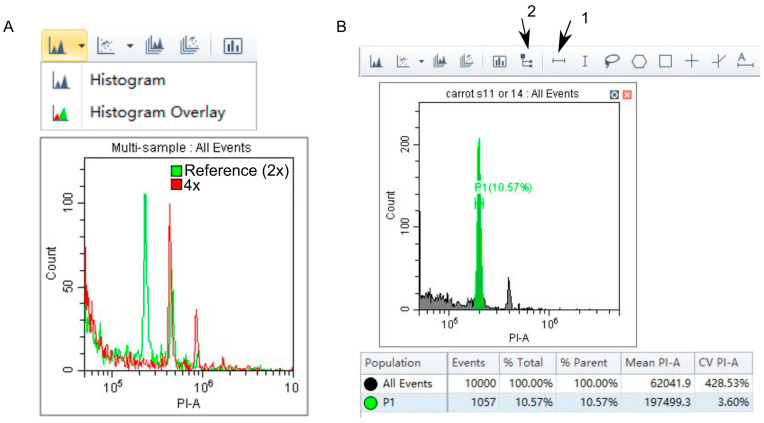
Flow cytometry data analysis. (**A**). The ploidy of summer squash (*Cucurbita pepo L. subsp. pepo*) regenerant produced by doubled haploid technology was analyzed. Seed-derived summer squash leaves were used as a reference standard. Nuclei were isolated by razor chopping in Galbraith buffer and stained with PI. Reference standard (2x) histogram (green) was overlaid with the regenerant histogram (red). The regenerant G_0_/G_1_ peak overlays with reference G_2_ peak. Hence, the regenerant is a tetraploid plant (4x). (**B**). Create G_0_/G_1_ peak population (P1) using the tool (1). Create statistics window (shown below the plot) using the tool (2). Click on statistics window, pick mean and CV values to be displayed.

This can be done by switching from acquisition to analysis screen and going to histogram overlay. Drag and drop samples from the list into the histogram overlay window (Figure 2A). If this function is not available in the cytometry program or the instrument usage time is limited, use a specialized flow cytometry software on a separate computer. Flowing software can be used (Figure S1), since it is free and extended instructions are available online.

- Export results or print them to a pdf file.

#### 3.4.2. DNA Ploidy/Content Quantifications

- Select G_0_/G_1_ peaks at the acquisition screen for the reference standard (same or separate plot for internal and external reference standard, respectively) and analyzed sample (Figure 2B).
- Create statistics window (Figure 2B). Click on statistics window and add mean and CV to displayed parameters. This can also be done using Flowing software Statistics function (Figure S1).

The desired CV values are below 3%. CV values exceeding 5% are considered unacceptable, but sometimes lower CV cannot be achieved because of sample specifics (high phenolics, very small genome, herbarium used as a source of nuclei for analysis) [8].

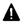
 CRITICAL STEP. To improve CV values, try different amounts of leaf tissue and different intensity of razor chopping. Use fresh sample with high turgor. Test different buffer and antioxidants. Deep clean and de-bubble the instrument.

- Calculate DNA ploidy or DNA content.

DNA content provides information on the amount of DNA, but not the number of chromosomes. The term “DNA ploidy” should be used for flow cytometry data instead of the term “ploidy”, unless DNA ploidy data are confirmed by conventional chromosome counting. A large DNA ploidy value does not mean that the organism has a lot of chromosomes, because it can have a small number of large chromosomes and vice versa [25].

DNA content and ploidy terms are complicated in plants because of widely spread polyploidy and other large scale genomic rearrangements. It is proposed to use the terms “monoploid” and ”holoploid” for clarity [26]. Monoploid genome (designated by x) contains one chromosome set. Holoploid genome (designated by n) is the meiotically reduced chromosome number regardless of generative polyploidy or aneuploidy [26]. DNA content of a holoploid (n) and monoploid (x) genome are designated by C-value and Cx-value, respectively. For full terminology refer to [26]. As an example, bread wheat, Triticum aestivum, is a hexaploid plant, thus we have 2n = 6x = 42 chromosomes and DNA content 2C = 6Cx [8].

Use the following formula for DNA content calculations:

(1) $$Sample\;ploidy\left(DNA\;content\right)]\;=Reference\;ploidy\left(DNA\;content\right)\;\times \frac{Sample\;mean\;of\;the\;G_{0}/G_{1}\;peak}{Reference\;mean\;of\;the\;G_{0}/G_{1}\;peak}$$

Reference standard plant C- or 2C-value is usually used for DNA content calculations.

- Obtain DNA content values from three measurements performed on different days.
- Calculate mean and SD from three measurements.

The difference between minimum and maximum value between measurements should not exceed 2% [8].

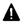
 CRITICAL STEP. If unexpected ploidy or DNA content results are observed or the same samples give different results (more than 2% difference) on the same or different days, the results must be treated with caution. The instrument deep clean and/or de-bubbling should be performed. Antioxidant addition should be tried. If results are reproducible, unexpected ploidy results and ploidy variability in different tissues of the same plant should be confirmed with microscopic analysis of samples stained for DNA.
- Compare DNA content results to published C-values available in Plant DNA C-values Database [27].

## 4. Conclusions

Starting flow cytometry in the lab requires extended knowledge of flow cytometer setup and management. Unique plant anatomy and biochemistry also pose considerable challenges to researchers. However, solving a multifactor problem of obtaining quality cytometric data pays off with the acquisition of DNA content data for a large number of samples in a short period of time. DNA content data can provide invaluable insight into applied and fundamental questions of plant sciences.

## Data Availability

Not applicable.

## Supplementary Materials

The following supporting information can be downloaded at: https://www.mdpi.com/article/10.3390/mps6010018/s1, Figure S1: Flowing software DNA content analysis workflow; Table S1: Otto I buffer recipe; Table S2: Otto II buffer recipe; Table S3: LB01 buffer; Table S4: Galbraith buffer; Table S5: Tris-MgCl2 buffer; Table S6: A variant of complete lysis buffer.
