# Supplementary material for: Mastering DNA Content Estimation by Flow Cytometry as an Efficient Tool for Plant Breeding and Biodiversity Research
Source: Methods Protoc. 2023 Feb 12;6(1):18. doi: 10.3390/mps6010018 (PMC9963313; doi:10.3390/mps6010018)
Supplement: Supplementary file 1 [file mps-06-00018-s001.zip › mps-2120567-supplementary.pdf]

Supplementary material

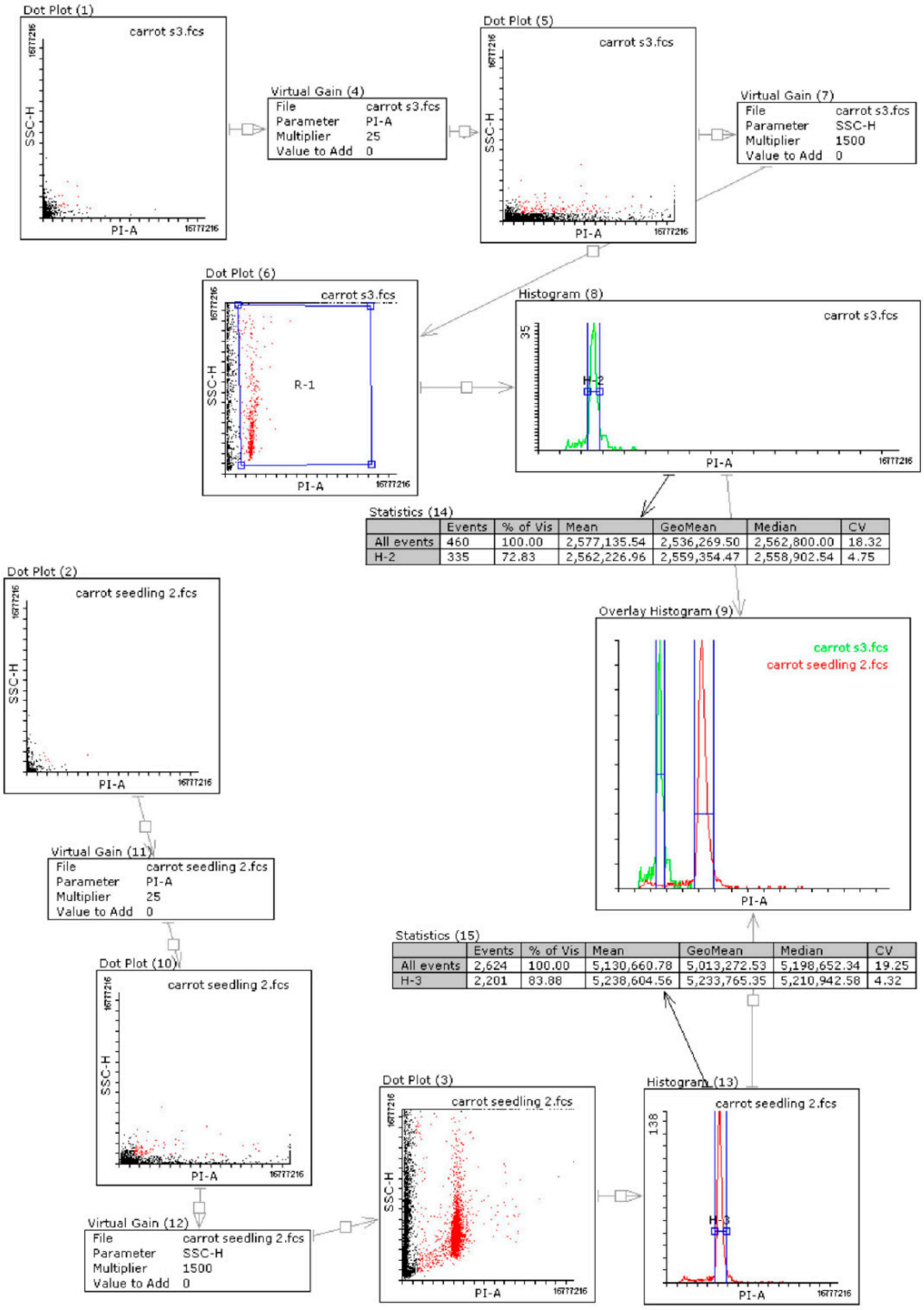

Figure S1. Flowing software DNA content analysis workflow.

Nuclei from control carrot seedlings (2x) and regenerants produced by doubled haploid technology were isolated and stained with PI. Regenerant data (dot plot (1)) were stretched along X and Y axis (Virtual gain (4) and (7), respectively) to produce Dot plot (6). Nucleus population was manually gated (R1 gate). Histogram (8) is produced to visualize nuclear peak. The peak was manually gated (H2). Statistics table with Mean and CV data was produced. The same was performed for control sample (dot plot (2)). Control and regenerant sample histograms were overlayed for better visualization (Overlay histogram (9)). Regenerant ploidy = Control ploidy × $Mean_{Regenerant}$ / $Mean_{control}$ = 2n × 2562226,96 / 5238604,56 = 0.978n. Hence, the regenerant plant is haploid.

Table S1. Otto I buffer recipe [23,24]. It is used for the nucleus isolation step.

Component	Final concentration	Amount to add
Citric acid	0.1 M	2.58 g
Tween 20	0.5%	500 μL
H_2O		Up to 100 mL

pH 2-3, no adjustment needed

Table S2. Otto II buffer recipe [23,24]. It is added to Otto I buffer with isolated nuclei for DNA staining step (DNA dye is added to Otto II or to the mixture of Otto buffers).

Component	Final concentration	Amount to add
Na_2HPO_4 x 12 H_2O	0.4 M	5.68 g
H_2O		Up to 100 mL

pH 8-9, no adjustment needed

Table S3. LB01 buffer [25].

Component	Final concentration	Amount to add
Tris	15 mM	0.1815 g
Na_2EDTA	2 mM	0.0584 g
Spermine 4HCl	0.5 mM	0.0174 g
KCl	80 mM	0.6 g
NaCl	20 mM	0.116 g
Triton X-100	0.1%	100 μL
H2O		Up to 100 mL

pH 8.0

Table S4. Galbraith buffer [6].

Component	Final concentration	Amount to add
$MgCl_2$	45 mM	0.045 g
MOPS	20 mM	0.418 g
Sodium citrate	30 mM	0.774 g
Triton X-100	0.1%	100 μL
H_2O		Up to 100 mL

pH 7.0

Table S5. Tris-$MgCl_2$ buffer [26].

Component	Final concentration	Amount to add
Tris	200 mM	2.42 g
$MgCl_2$ x $6H_2O$	4 mM	0.038 g
Triton X-100	0.5%	500 μL

H_2O		Up to 100 mL

pH 7.5

Table S6. A variant of complete lysis buffer (prepared immediately before sample preparation)

Component	Final concentration	Amount to add
Ascorbic acid (stock solution 100 mg/mL in water)	0.2 mg/mL	20 µL
RNase I (10 µg/mL)	50 µg/mL	50 µL (1:200)
PI (stock solution 1 mg/mL in water)	50 µg/mL	500 µL (1:20)
Ice cold buffer		Up to 10 mL
